# Formulation Optimization of Sucrose-Free Hard Candy Fortified with *Cudrania tricuspidata* Extract

**DOI:** 10.3390/foods10102464

**Published:** 2021-10-15

**Authors:** Yoowha Jeon, Jieun Oh, Mi Sook Cho

**Affiliations:** 1 Department of Nutritional Science and Food Management, Ewha Womans University, Seoul 03760, Korea; gonggang4039@naver.com; 2 College of Science and Industry Convergence, Ewha Womans University, Seoul 03760, Korea; oje96@ewha.ac.kr

**Keywords:** confectionery, sugar-free, D-optimal, optimization

## Abstract

The aim of the study is to define the optimal formulation of sucrose-free hard candy using D-optimal mixture design as the base for the incorporation of *Cudrania tricupidata* fruit. Hard candy was produced using three different polyols: isomalt, maltitol syrup, and xylitol. This study examined the effects of polyol mixtures as sucrose and corn syrup substitutes on physicochemical (moisture, color, soluble solid (SSC)), hardness, and sensory features of hard candies. These three polyols had notable effects on quality characteristics in addition to their effects on L* value. Xylitol had an undesirable effect on moisture content and hardness, resulting in decreased texture acceptability, but improved color and clarity. Given the results of our experiments and optimization of variables, we determined that 90.21% isomalt, 8.63% maltitol syrup, and 1.16% xylitol produced a sugar-free candy with high desirability (0.894).

## 1. Introduction

Today’s consumers demand naturally derived food ingredients [1,2], due to the legislative restrictions and consumer concerns about the use of synthetic additives [3,4]. As the potential toxicologic effects of the use of synthetic food colorants on human health have been indicated [5,6,7], the food industry has been searching for natural ingredients containing both visual pigments and bioactive materials in place of artificial food dyes [8,9,10]. This is because the incorporation of these natural ingredients can not only improve the nutritional quality of food products but add naturalness and sensory acceptance [11,12,13]. Accordingly, there is a growing interest and research regarding the application of natural ingredients in food, confectionery, and beverages [14,15].

*Cudrania tricuspidata* is a tree in the mulberry family that is widespread throughout East Asia [16]. It contains abundant xanthones and flavonoids along with organic acids and has been shown to have antioxidant, anti-inflammatory, anti-tumor, and hepatic protective properties [16,17,18,19,20]. Among the different components of *Cudrania tricuspidata*, the mature fruit has a sweet taste due to its high sugar content (17.73 Brix) [21,22] and vivid red color owing to carotenoid pigments (redder than the Korean raspberry, Rubus coreanus) [21]. Several studies to investigate the effects of *Cudrania tricuspidata* fruit fortification on foods indicated that color parameters significantly depend on the level of this fruit [23,24,25]. Given that the use of fruit in confectionery products can replace artificial colorants usually used in traditional products [26,27], *Cudrania tricuspidata* fruit can be a good natural additive that confers the desired color and simultaneously improve the nutritional profile of confectionery products.

With growing consumer awareness of the health effects of sugar overconsumption, sugar-free products have become popular in the food industry, especially in sugar confectionery. The increase in consumer demand for sugar-free products that are comparable to sugar counterparts [28] has spurred food science research into alternative sweeteners that have a similar sensory profile to sucrose. Although sucrose substitutes have commercial advantages, such as low caloric value [29], they have limitations with regard to imitating the sensory and physical attributes of sucrose, such as clean taste and texture [30]. It is thus challenging to produce acceptable sugar-free products given that all of the sucrose in the product needs to be replaced [31]. Kilara [32] reported that blending different polyols has various advantages (i.e., desired sensory attributes, process stability, shelf-life stability, and cost) in ice cream and frozen desserts, and these polyol blends can be used as sweeteners in the production of hard candy [33] due to their stability [34]. Although polyols have low solubility and viscosity in addition to low sweetening power in hard candy applications [35], a recent study demonstrated that replacing 15–30% isomalt with a hydrogenated starch hydrolysate (HSH) such as maltitol syrup increased solubility and viscosity [35] and had a synergistic sweetening effect. Likewise, xylitol can improve sweetness when combined with isomalt [36]. These findings indicate that maltitol syrup and xylitol used in combination may facilitate the development of a highly palatable isomalt-based hard candy. Considering that the proportions of polyols in the mixture are dependent on each other and their sum is always 100%, mixture designs are appropriate to investigate the effects of components of food products on the final product and identify component interactions [37,38,39]. Amongst several mixture designs, a D-optimal mixture design can be used to determine the optimal formulae of products containing various ingredients with high prediction accuracy [40]. This technique has recently been utilized in food research that optimizes product components, such as hard candy [41], chewing gums [42], and milk tablets [43].

Findings from previous studies illustrated that the combination of polyols influences quality characteristics and sensory properties in confectionery, including chewy candy [44], and chocolates [45,46,47,48]. Unfortunately, research to examine polyol interaction in hard candy systems is limited, despite widespread consumption. When it comes to sucrose-free hard candy products, most of them are made with one type of polyol and one or two high-intensity sweeteners such as sucralose and acesulfame-K to enhance the sweetness. However, there has been little research on the long-term adverse health effects of consuming high-intensity sweeteners [49]. It is necessary to investigate how polyol mixtures affect the quality parameters of candy in order to develop sucrose-free hard candy made only of polyols with high sensorial acceptance. Thus, the present study was aimed to examine the effect of isomalt, maltitol syrup, xylitol, and their interactions on the properties of candy and to define the best formulation. *Cudrania tricuspidate* fruit extract was added as a replacement for artificial coloring additives.

## 2. Materials and Methods

### 2.1. Materials

The following materials were used for production of hard candies:

- Natural flavorings: *Cudrania tricuspidata* fruit extract (prepared by decoction according to Section 2.3.1); 65 Brix lemon extract (Serim Food, Bucheon, Korea); 64 Brix ginger extract (ES Food, Seoul, Korea).
- Sucrose-free hard candy: isomalt (Palatinit Subungsmittel GmbH, Mannheim,, Germany); maltitol syrup (Samyangcorp quone, Korea); xylitol (Danisco Sweeteners Oy, Sokeritehtaantie, Finland); water.
- Traditional hard candy (control): refined granulated sucrose (Samyangcorp quone, Seoul, Korea); 70 DE glucose syrup (Ottogi, Seoul, Korea); water.

### 2.2. Experimental Design

**Figure 1:**
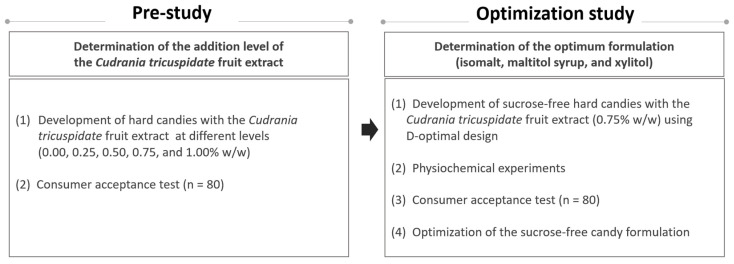
Flow chart of the research process.

The current study was designed to (1) determine the most suitable addition level of *Cudrania tricuspidata* fruit extract in hard candy (2) and to define the optimal conditions for the formulation of sugar-free hard candy with *Cudrania tricuspidata* fruit extract. This is in accordance with ethical research on human beings and was approved by the Ethical Committee of Ewha Womans University (ewha-2020080005-03). The comprehensive experimental design is shown by flow chart (Figure 1).

#### 2.2.1. Determination of the Addition Level of *Cudrania tricuspidata* Fruit Extract

A 9-point hedonic preference scale was performed to determine the most acceptable addition level of *Cudrania tricuspidata* fruit extract in hard candy. For this study, traditional hard candy, prepared from sucrose and glucose syrup, was chosen as a food matrix. The candy samples were manufactured by using 5 different levels of the extract (0.00%, 0.25%. 0.51%, 0.75%, 1.00%) (see Section 2.3.1 and Table 1).

A total of 80 female consumers, ranging from 20 to 29 years of age, participated. The acceptance test was conducted in private booths under white fluorescent lights for each panelist. They were asked to evaluate the samples for overall acceptance, followed by appearance, taste, texture, color, clarity, sweetness, sourness, refreshing odor, hardness, fracturability, and stickiness, using a 9-point hedonic scale (dislike extremely to like extremely).

#### 2.2.2. Determination of Sugar-Free Candy Formulation by D-Optimal Mixture Design

The Design-Expert 12.0 (Stat-Ease Inc., Minneapolis, MN, USA) software was utilized to determine the optimal proportions of the sucrose-free hard candy formulation. A D-optimal mixture design was employed to evaluate the effects of isomalt (A), maltitol syrup (B), xylitol (C) on the physicochemical and sensory properties of candy samples and determine the optimum mixture. The three component ranges were as follows: isomalt (A: 50–100%), maltitol syrup (B: 0–40%), xylitol (C: 0–15%). The Design-Expert designed 12 runs which eight runs were different, and two runs were replica runs. The proportions of polyols were indicated as fractions of the mixture with a sum (A + B + C) of 98.45. Since the pre-study to determine the addition level of *Cudrania tricuspidata* fruit extract in candy showed that candy with 0.75% of the extract had the highest overall acceptability score, the present study decided to add 0.75% of the extract to each formulation. The mixture design and the amount of ingredients are expressed in Table 2.

### 2.3. Sample Preparation

#### 2.3.1. Manufacturing of *Cudrania tricuspidata* Fruit Extract

The *Cudrania tricuspidata* fruit extract was prepared based on the method of Ji and Jeong [50]. Locally purchased frozen fruits of *Cudrania tricuspidata* were used to produce the extract. Berries were thawed at room temperature (25 °C) for 12 h and blended with 200 mL of distilled water using a mechanical blender (EZ600, Blendtec, Orem, USA) for 2 min. The mixed mass was filtered using a 20-mesh sieve and fine filtering cloth (1). The residue was also blended with 200 mL of distilled water and filtered the same way (2). One hundred milliliters of distilled water were added to the second remnant and blended and filtered as in step 2. (3). Juices generated from the three steps (1 + 2 + 3) were boiled in a ceramic coating pot at 80 °C (905-T1, Testo, Lenzkirch, Germany) for 4 h. The total amount of the solid ingredients remaining after filtration was 410.3 g and that of the filtrate before boiling was 1905.9 g. As a result, 334 g of extract was obtained and stored in the refrigerator until the preparation of hard candy.

#### 2.3.2. Preparation of Traditional Hard Candies

**Figure 2:**
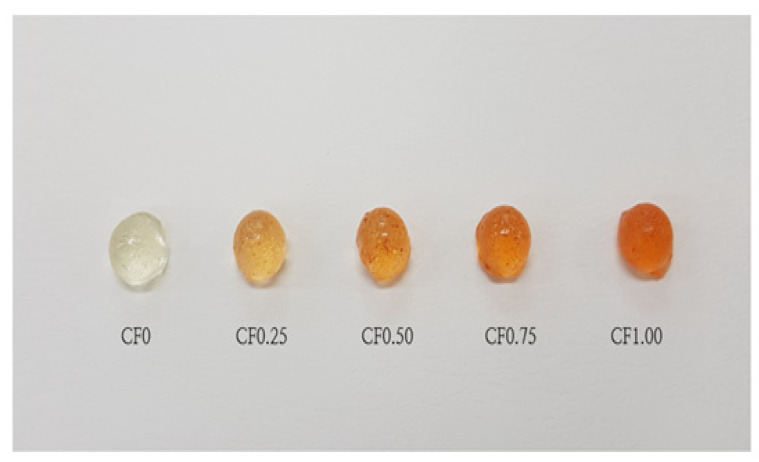
Hard candy sample with added Cudrania tricuspidate fruit extract.

Traditional hard candy samples (five formulations) were produced by open fire cooking [51] based on the procedure of hard candy manufacturing described by Bunce [52] and Hartel et al. [53]. Samples were formulated with sucrose: glucose syrup (40.00%). According to Hartel et al. [53] it is common to add 1.0–1.5% of fruit extract in the manufacturing of hard candy. As *Cudrania tricuspidata* fruit extract has relatively high moisture content, this extract was set from 0.00% to 1.00%. Ginger extract, lemon extract and mint flavoring were added as well for the sake of improving the candy flavor. The formula of five candy samples was specified in Table 1. Sucrose and glucose syrup were first dissolved in water and heated without stirring up to 160 °C (905-T1, Testo, Germany). When the temperature of the mixture reached 160 °C, the candy paste was cooled. At 112–115 °C, *Cudrania tricuspidata* fruit, lemon and ginger extracts, and mint flavoring were added to the mass and mixed until a homogeneous mixture was obtained. The final mixture was poured into silicone molds and cooled down at 20 °C for 5 min. Solid candies removed from the molds were packed in aluminum bags and stored in a refrigerator (10 °C) until required for analysis. The final samples are shown in Figure 2.

#### 2.3.3. Preparation of Sucrose-Free Hard Candies

**Figure 3:**
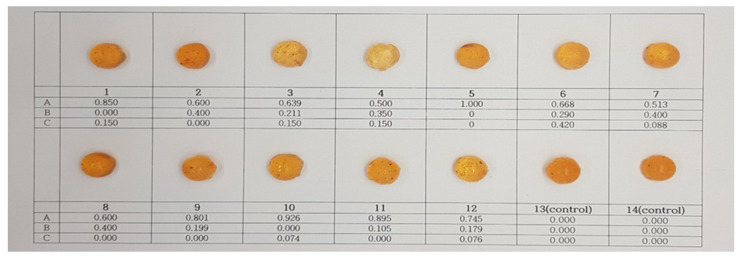
Sucrose-free hard candies with *Cudrania tricuspidate* fruit extract. A: Isomalt, B: Maltiol syrup, C: Xylitol.

Open fire cooking was also conducted to prepare sucrose-free hard candies. The 12 samples were formulated according to the mixture design (Table 2) with the following ingredients: polyols (98.45%), *Cudrania tricuspidata* fruit extract (0.75%), lemon extract, ginger extract, mint flavoring, and water; the additional level of *Cudrania tricuspidate* fruit extract (0.75%) was determined by precursory sensory evaluation. Dissolved polyols in water were heated until a temperature of 170 °C was reached, and the mixture was then cooled. At 112–115 °C, the mass was blended with *Cudrania tricuspidata* fruit, lemon and ginger extracts, and mint flavoring. Afterward, candies were molded, packed, and stored in the same way as the traditional hard candy samples. These candy samples are shown in Figure 3.

### 2.4. Physicochemical Analysis

Moisture content was measured using a halogen moisture analyzer (MX-50, A&D Company, Tokyo, Japan). Soluble solid content (SSC) determination was conducted with a refractometer (SCM-1000, HM DIGITAL, Seoul, Korea) and values are expressed in Brix. Values are mean values from three replicate experiments.

### 2.5. Color Analysis

Hunter color scale values of candy samples, L* (brightness), a* (redness), b* (yellowness), were determined using a spectrophotometer (CM-500D, Konica Minolta, Tokyo, Japan) according to the method of Lee et al. [54]. Mean values were determined from three replicate measurements.

### 2.6. Hardness Analysis

The hardness of candy formulations was determined using a texture analyzer (TAXT2i, Stable Microsystems Ltd., London, UK) with a load cell weight of 50 kg and a penetrating probe (needle P/2). Samples were compressed to 50% of their original height at a test speed of 1 mm/s. Hardness values are expressed as kilogram-force (kgF). Ten replicate assessments were performed and mean values of these ten values are reported.

### 2.7. Sensory Analysis

A total of 80 female consumers in their 20s, who had not participated in the precursory acceptance test, took part in the sensory evaluation. The assessment was conducted in two sessions, which took place on different days to avoid tiredness. Seven candy samples were randomly presented in white plastic dishes per session. Sessions were performed in individual booths under fluorescent light. The panel evaluated each sample, coded with random three-digit numbers, regarding overall acceptance, appearance, taste, texture, color, clarity, sweetness, sourness, refreshing odor, hardness, fracturability, and stickiness using a 9-point hedonic scale from 1 (extremely dislike) to 9 (extremely like). Consumers were requested to rinse their mouths with deionized water and crackers between evaluations [55].

### 2.8. Statistical Analysis

Analysis of variance (ANOVA) was performed to analyze parametric data in the precursory acceptance test to determine an appropriate addition level of *Cudrania tricuspidate* fruit extract, using IBM SPSS Statistics for Windows, version 22.0 (IBM Corp, NY, USA, 2013). Mean differences were identified by Duncan’s post hoc test at a significance level of 0.05.

In the following study, the analysis of experimental data was conducted by multiple regressions to fit the polynomial equation to all independent variables. Linear, cubic, quadratic, and special quartic models were utilized to fit response values (Equations (1)–(4)). To assess the statistical significance of each equation, ANOVA at *p* < 0.05 was used. Design-Expert 12.0 (Stat-Ease Inc., Minneapolis, MN, USA) software was utilized to carry out all computational work including predicted equation, analysis of variance, fitting for the best model, model validation, and visualization of contour plots.

(1) Y=λ1X1+λ2X2+λ3X3 (linear)

(2) Y=λ1X1+λ2X2+λ3X3+λ1λ2X1X2+λ1λ3X1X3+λ2λ3X2X3 (quadratic)

(3) Y=λ1X1+λ2X2+λ3X3+λ1λ2X1X2+λ1λ3X1X3+λ2λ3X2X3+ λ1λ2λ3X1X2X3 (cubic)

(4) $$Y=\lambda 1X1+\lambda 2X2+\lambda 3X3+\lambda 1\lambda 2X1X2+\lambda 1\lambda 3X1X3+\lambda 2\lambda 3X2X3+\lambda 1^{2}\lambda 2\lambda 3X1^{2}X2X3+\;\;\lambda 1\lambda 2^{2}\lambda 3X1X2^{2}X3+\;\lambda 1\lambda 2\lambda 3^{2}X1X2X3^{2}\;(\mathrm{special}\;\mathrm{quartic})$$

Y is the response variable, i.e., physicochemical properties (moisture content, soluble solid content), color (L*, a*, b*), hardness, and sensory assessment parameters (overall acceptance, appearance, flavor, texture, color, clarity, sweetness, sourness, cooling, hardness, fracturability and stickiness). λ represents the constant coefficients for linear and non-linear terms. The significant regression coefficients (*p* < 0.05) are bold in the following equations in Section 3.1.1.

## 3. Results and Discussion

### 3.1. Optimization Study

Values for responses (moisture, SSC, L* (brightness), a* (redness), b* (yellowness), hardness) are expressed as means ± standard error of the mean (SEM) in Table 3. The results of the sensory evaluation are presented in Table 4.

A best-fit model should have a low standard deviation and predicted sum of squares and high R-squared value [56,57]. In the current study, we selected models with the highest R-squared values and p-values below 0.05 as the best-fit models [45]. The effects of three polyols on physicochemical properties, color parameters, hardness parameter and sensory properties are reported by the coefficient of the adjusted model.

Regarding experimental parameters, a linear model was chosen for moisture (b*) and hardness while a quadratic model was selected as the best-fit model for SSC. No acceptable model was determined for L* and a*.

For sensory parameters, a linear model was adopted for overall liking, texture, clarity, hardness, fracturability, and tooth stickiness. A special quartic model was selected for color while a cubic model best depicted cooling. However, no statistical models for appearance, flavor, sweetness, or sourness were found.

#### 3.1.1. Physicochemical Properties

The moisture content of candy samples ranged from 3.05 g/100 g to 5.46 g/100 g. The effects of isomalt (A), maltitol syrup (B), and xylitol (C) on moisture content response are summarized in regression Equation (5):

Moisture = 2.90A + 3.88B + 6.39C(5)

**Figure 4:**
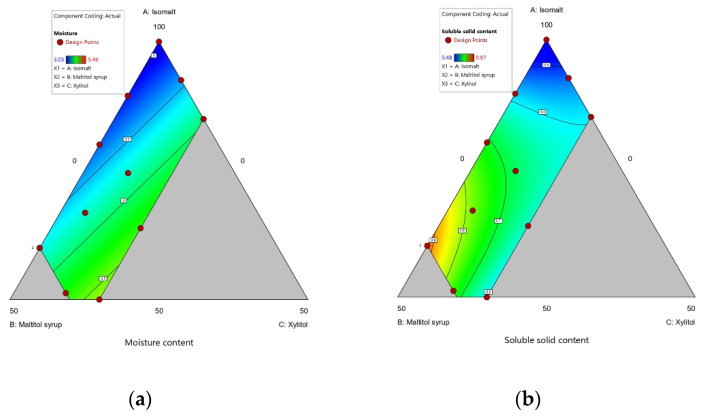
(**a**) Contour plot of moisture content; (**b**) Contour of soluble solid content. (A: Isomalt, B: Maltitol syrup, C: Xylitol).

All samples except for sample 4 had a moisture content within the range of that of commercial hard candies, namely less than 5% *w*/*w* [58]. Moisture content increased significantly as xylitol level increased (Table 3 and Figure 4). A similar result was reported for the preparation of sucrose-free milk chocolate with isomalt, xylitol, and maltitol using a simplex lattice design [46]. This can be explained by the high hygroscopicity of xylitol [59]; xylitol molecules have a high number of active hydroxyl groups (-OH) that bond with water molecules compared to other sweeteners [60]. Hence, xylitol-containing candies are more hygroscopic and have a relatively high moisture content, which can generate a stickiness problem [61].

In addition, the majority of sucrose-free candy samples had a higher moisture content than those of the control samples (Table 3 and Figure 4). Aidoo and Afoakwa [62] reported that a higher moisture level was observed with a high amount of sucrose substitutes. Given that control candies contained 40% glucose syrup, a hygroscopic material, and were prepared at a lower temperature than sucrose-free samples, sucrose may absorb less water from the air than other sweeteners, in the context of hard candies.

The soluble solid content (SSC) of candies ranged between 0.48 and 0.97 Brix. The regression equation for the SSC response influenced by independent variables is indicated in Equation (6):

SSC = 0.4158A + 0.9967B + 0.2180C + 0.2568AB + 1.14AC − 0.7937BC(6)

Results indicate that SSC was strongly influenced by the type of polyol. A higher level of maltitol syrup increased SSC values, whereas isomalt and xylitol had the opposite effect. This is because maltitol syrup is in liquid form and has a higher solubility than other polyols [35]. However, xylitol is also highly soluble in water [63], but xylitol did not increase the SSC of the hard candy samples. This may be because only a limited amount of xylitol (0–15%) was added in this study.

#### 3.1.2. Color

Food color affects consumer acceptability [64], so we examined whether the color of candies was affected by different compositions of polyols. L* (brightness), a* (redness), b* (yellowness) values of samples ranged from 55.10–63.77, 21.29–25.86, and 31.60–40.05, respectively. Significant models to explain the effects of polyols on color parameters were determined for a* and b*, and are shown in Equations (7) and (8):

a* = 26.07A + 26.83B + 44.06C − 16.97AB − 27.84AC − 51.54BC − 153.84A2BC + 553.09AB2C − 16.32ABC2(7)

b* = 38.25A + 37.59B + 23.97C(8)

**Figure 5:**
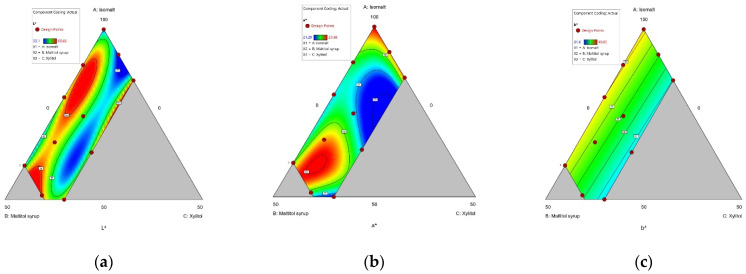
(**a**) Contour plot of L* (brightness); (**b**) Contour of a* (redness); (**c**) Contour of b* (yellowness). (A: Isomalt, B: Maltitol syrup, C: Xylitol).

The use of xylitol resulted in a significant decrease in yellowness value, as opposed to isomalt and maltitol syrup (Figure 5). Candy formulations containing xylitol had a lower redness value except for sample 1 (Table 3). In addition, high brightness values were found in samples with xylitol compared to samples consisting of isomalt and maltitol syrup, and the control (Table 3). However, previous studies that examined the effects of polyols on the color of sugar confectionery, including sucrose-free chocolates [45,46] and marmalade [65], reported lower brightness values for samples containing 100% xylitol. Our findings and those of previous studies [45,55] suggest that changes in color parameters are likely to be due to interactions between different types of polyols, resulting in physicochemical changes (e.g., in solubility), as well as interactions between the pigment of *Cudrania tricuspidata* fruit extract and the polyols.

#### 3.1.3. Hardness

There were significant differences in hardness between candy samples. Hardness values ranged from 11.17 to 29.62 depending on the concentrations of isomalt, maltitol syrup, and xylitol. The impact of independent variables on hardness response is expressed by Equation (9):

Hardness = 29.38A + 20.75B + 5.11C(9)

**Figure 6:**
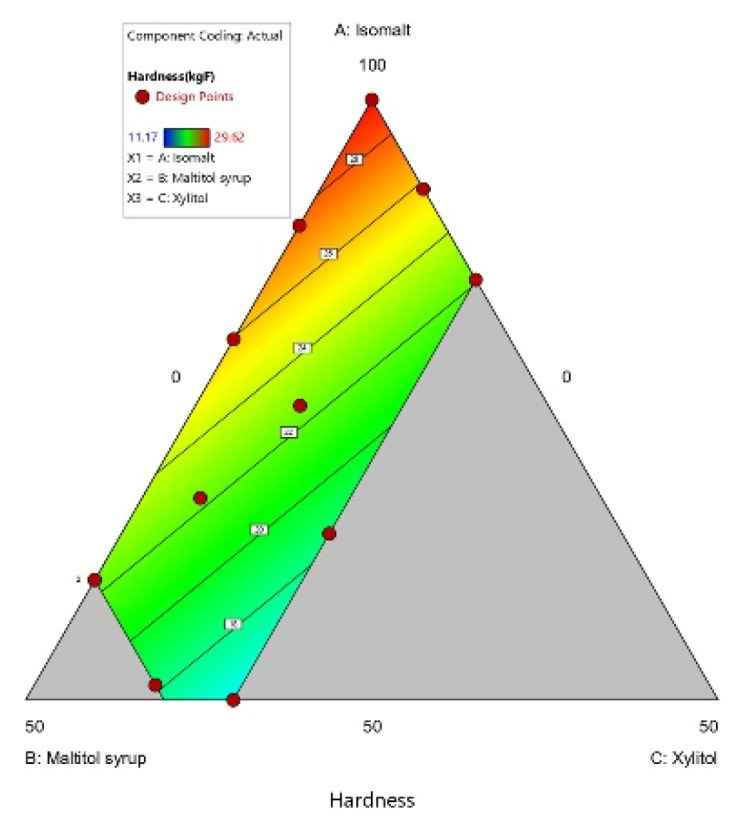
Contour of Hardness. (A: Isomalt, B: Maltitol syrup, C: Xylitol).

Candy hardness was found to increase with an increase in the level of isomalt, but decrease with an increase in the level of maltitol syrup and xylitol (Figure 6). The formulation containing 89.5% isomalt and 10.5% maltitol syrup (sample 11) had the highest hardness value. However, candies comprising 60% isomalt and 40% maltitol syrup (samples 2, 8) had relatively low hardness values. As molecular interactions have a significant effect on hardness, further studies are needed to determine how interactions between isomalt and maltitol syrup differ according to their blending ratio. Meanwhile, formulations containing all three components had relatively low hardness values amongst the 14 candy samples (Table 3). Xylitol increased hygroscopicity and the stickiness of hard candy when combined with other polyols [66]. However, a significant difference was found in hardness values among samples 1, 3, and 4, even though each of these samples contained the same amount of xylitol (15%). Given that sample 1, with the highest amount of isomalt, had the highest hardness value of these three candy samples, it appears that a higher concentration of isomalt can reduce some of the negative effects of xylitol, leading to good hardness in candy containing xylitol.

#### 3.1.4. Sensory Properties

Because consumers demand reduced sucrose/sucrose-free products with similar sensory properties as conventional ones [67], consumer satisfaction is a key goal in the development of food products containing sucrose substitutes [28]. Therefore, it is essential to conduct sensory analysis in food reformulation [68].

We investigated the appearance, flavor, texture, color, clarity, sourness, sweetness, cooling, hardness, fracturability, stickiness, and overall acceptability of hard candy samples using a 9-point hedonic scale. No significant models were found for appearance, flavor, sourness, or sweetness at *p* <0.05. Significant models were found for the following sensory properties (Equations (10)–(17)):

Texture = 6.65A + 5.96B − 2.01C(10)

Color = 5.98A + 5.97B + 3.36C + 0.002AB + 4.36AC + 4.37BC +23.43A2BC − 40.09AB2C + 19.52ABC2(11)

Clarity = 5.71A + 6.08B + 6.92C(12)

Cooling = 5.45A + 8.96B + 117.89C − 8.69AB − 204.31AC − 203.97BC + 221.77ABC + 12.34AB(A−B) + 107.29AC(A−C) + 79.49BC(B−C)(13)

Hardness = 6.44A + 5.71B − 3.28C(14)

Fracturability = 6.48A + 5.98B − 5.34C(15)

Stickiness = 6.41A + 5.63B − 5.30C(16)

Overall acceptance = 5.94A + 5.85B + 0.3270C(17)

**Figure 7:**
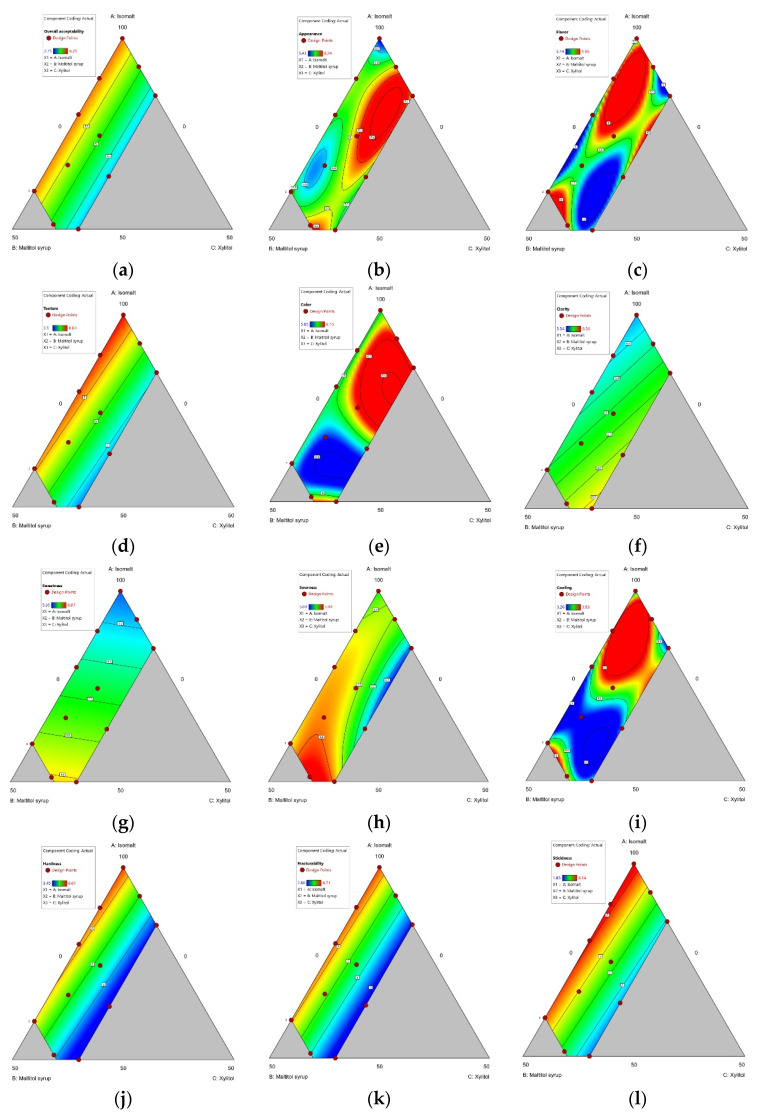
(**a**) Contour plot of overall acceptability; (**b**) Contour of appearance; (**c**) Contour of flavor; (**d**) Contour of texture; (**e**) Contour of color; (**f**) Contour of clarity; (**g**) Contour ofsweetness; (**h**) Contour ofsourness; (**i**) Contour ofcooling; (**j**) Contour of fracturability; (**k**) Contour of hardness; (**l**) Contour of stickiness. (A: Isomalt, B: Maltitol syrup, C: Xylitol).

According to the results of sensory evaluation, interactions among the three components negatively influenced the overall acceptability of hard candy and sensory texture properties (Figure 7). This can be explained by the ability of xylitol to increase sorption capacity when mixed with other polyols in hard candy [66]. Physicochemical analysis identified that the moisture content of candy increased, and its hardness decreased with an increase in xylitol combined with maltitol syrup. An increase in moisture content of hard candy results in a softer texture, sticky surface, and increased tooth stickiness [58], all of which decrease overall acceptability [69]. The present study also found that these undesirable characteristics led to lower satisfaction with the general sensory properties of the hard candy.

The interaction between two and/or three sweeteners had a positive effect on visual sensory properties, color, and clarity (Figure 7). Instrumental color results indicated that xylitol significantly lowered the a* and b* values of candies as opposed to 100% isomalt candy (sample 5). As these color values were markedly lower in samples prepared with three polyols than in isomalt-based candies with only one variable (sample 1, 2, 5, 8, 10, 11) (Table 4), the interactivity between maltitol syrup and xylitol appears to affect the solubility of the colorant, thereby increasing the transparency of the candy. As formulations comprising three polyols received higher liking scores for color and clarity, clearer candies with lower a* and b* values containing *Cudrania tricuspidata* fruit extract were considered more pleasant by consumers in our evaluation tests.

Sweetness acceptance was also higher for mixtures containing maltitol syrup and/or xylitol (Figure 7). Because both sweeteners not only have high solubility [35,70] but can interact synergistically with isomalt to increase sweetness [71], their combination resulted in hard candies with higher sweetness perception. However, no synergistic effect was found in the interaction between two and three elements with respect to refreshing aroma. Perceived aroma intensity decreases with an increase in the hardness in candies [72]. The highest refreshing aroma acceptance was observed for controls, which had a relatively low level of hardness among the 14 samples in the sensory evaluation. Taking this fact into account, the use of maltitol syrup and xylitol may decrease the release of aroma by the candy matrix, which leads to a decrease in acceptance of the candy.

#### 3.1.5. Hard Candy Formulation Optimization

**Figure 8:**
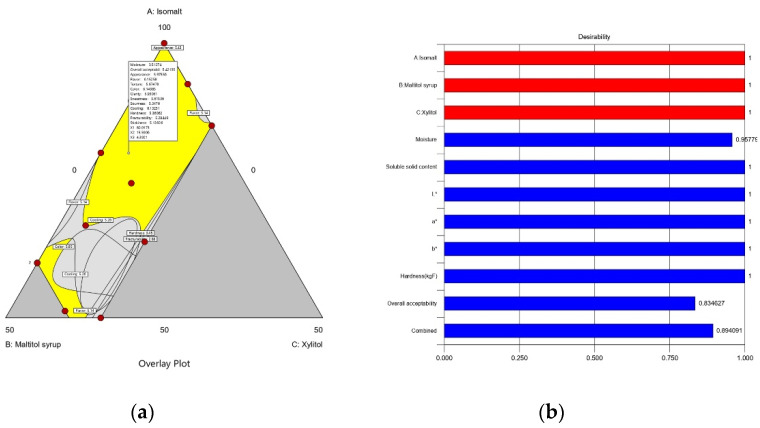
(**a**) Overlay of contour plot of physicochemical, color, hardness, and sensory acceptability for sucrose-free hard candies with Curania tricuspoidata fruit extract; (**b**) Desirability plot for optimum formulation.

To obtain sucrose-free hard candy with high palatability, optimization was conducted using software. Response values including soluble solid content, color, and hardness (19.47–27.33, according to texture acceptability) were set as “in range” whereas moisture content was set to “minimum”, and overall acceptability was set to “maximum”. The formulation of 90.21% isomalt, 8.63% maltitol syrup, and 1.16% xylitol had a desirability of 0.894 based on moisture, soluble solid content, color, hardness, and overall acceptability, as shown in Figure 8. As a product with a desirability value ranging from 0.8 to 1.0 is of excellent quality [73], this formula has the potential to produce a sucrose-free hard candy that will satisfy consumer demand and expectations.

### 3.2. Precursory Sensory Evaluation

Table 5 summarizes the outcomes of sensory analyses of the samples. There were significant differences in most sensory properties except for flavor, sweetness, and sourness acceptance. An increase in extract concentration decreased appearance acceptability as well as color and clarity. However, a similar trend was not found for texture, hardness, fracturability, or stickiness acceptability. Given that candies that received higher scores for texture properties also had higher overall acceptability scores, the texture appears to strongly influence the sensory quality of hard candies. In summary, a hard candy containing 0.75% *w*/*w Cudrania tricuspidata* fruit extract achieved significantly higher texture, hardness, fracturability, refreshing odor, and overall liking scores than other treatments. Therefore, the current study used 0.75% *w*/*w* of extract in the optimization study.

## 4. Conclusions

The growing demand for sucrose-free confectionery products encourages the total replacement of sucrose and glucose syrup with alternative bulk sweeteners. Consequently, to determine sucrose-free confectionery with enhanced physical quality and sensory acceptance, this study formulated sucrose-free hard candies using isomalt, maltitol syrup, and xylitol mixtures. *Cudrania tricuspidata* fruit extract was additionally added to this sucrose-free candies as a natural colorant. The current research indicated that the physical qualities and sensory parameters were both affected by the type of sucrose substitutes and the levels. In particular, high levels of xylitol adversely affected the physicochemical characteristics of the hard candy due to its unfavorable effects on texture qualities, although it increased color and clarity acceptance. From the foregoing results, it can be concluded that the manufacture of a desirable sucrose-free hard candy is attainable through the blending of isomalt, maltitol syrup, and xylitol.

## Figures and Tables

**Table 1:** Formula for the preparation of hard candy with addition of Cudrania tricuspidate fruit extract.

Figure Ingredients (g)	Samples				
	CF0 ^1^	CF0.25 ^2^	CF0.50 ^3^	CF0.75 ^4^	CF1.00 ^5^
Glucose syrup	40	40	40	40	40
Sucrose	59.20	58.95	58.70	58.45	58.20
*Cudrania tricuspidata* fruit extract	0.00 (0.00%)	0.25 (0.25%)	0.50 (0.50%)	0.75 (0.75%)	1.00 (1.00%)
Ginger extract	0.30	0.30	0.30	0.30	0.30
Lemon extract	0.30	0.30	0.30	0.30	0.30
Mint flavoring	0.20	0.20	0.20	0.20	0.20
Total	100	100	100	100	100

^1^ Hard candy with added 0% of *Cudrania tricuspidata* fruit extract. ^2^ Hard candy with added 0.25% of *Cudrania tricuspidata* fruit extract. ^3^ Hard candy with added 0.50% of *Cudrania tricuspidata* fruit extract. ^4^ Hard candy added with added 0.75% of *Cudrania tricuspidata* fruit extract. ^5^ Hard candy with added 1.00% of *Cudrania tricuspidata* fruit extract.

**Table 2:** D-optimal design model and sample preparation.

Sample	UnCoded Values			Real Values			Sucrose	Glucose Syrup	*Cudrania tricuspidata* Fruit Extract	Ginger Extract	Lemon Extract	Mint Flavor	Water	Total
	A ^1^	B ^2^	C ^3^	Isomalt	Maltitol Syrup	Xylitol								
1	0.850	0	0.150	83.68	0	14.77	0	0	0.75	0.30	0.30	0.20	30	130
2	0.600	0.400	0	59.07	39.38	0	0	0	0.75	0.30	0.30	0.20	30	130
3	0.639	0.211	0.150	62.91	20.77	14.77	0	0	0.75	0.30	0.30	0.20	30	130
4	0.500	0.350	0.150	49.22	34.46	14.77	0	0	0.75	0.30	0.30	0.20	30	130
5	1.00	0	0	98.45	0	0	0	0	0.75	0.30	0.30	0.20	30	130
6	0.668	0.290	0.042	65.79	28.52	4.14	0	0	0.75	0.30	0.30	0.20	30	130
7	0.513	0.400	0.088	50.46	39.38	8.61	0	0	0.75	0.30	0.30	0.20	30	130
8	0.600	0.400	0	59.07	39.38	0	0	0	0.75	0.30	0.30	0.20	30	130
9	0.801	0.199	0	78.82	19.63	0	0	0	0.75	0.30	0.30	0.20	30	130
10	0.926	0	0.074	91.12	0	7.33	0	0	0.75	0.30	0.30	0.20	30	130
11	0.895	0.105	0	88.14	10.31	0	0	0	0.75	0.30	0.30	0.20	30	130
12	0.745	0.179	0.076	73.36	17.64	7.45	0	0	0.75	0.30	0.30	0.20	30	130
13 (control)	0	0	0	0	0	0	58.45	40	0.75	0.30	0.30	0.20	30	130
14 (control)	0	0	0	0	0	0	58.45	40	0.75	0.30	0.30	0.20	30	130

^1^ The proportion of isomalt (%). ^2^ The proportion of maltitol syrup (%). ^3^ The proportion of xylitol (%).

**Table 3:** Physicochemical, color and hardness properties and fitted model statistics of sucrose-free hard candies added with *Cudrania tricuspidata* fruit extract.

**Sample**	Moisture (g/100g)	SSC	L*	a*	b*	Hardness (kgF)
1	3.80±0.50	0.58±0.17	63.77±3.50	25.51±5.25	35.06±1.49	26.55±4.12
2	3.87±0.99	0.97±0.03	55.91±3.62	23.61±1.09	34.26±2.56	23.40±3.93
3	3.96±0.47	0.70±0.08	63.36±1.22	22.24±1.20	33.67±1.20	19.42±2.06
4	5.46±0.24	0.53±0.05	62.99±2.83	21.29±1.68	31.60±1.59	11.17±0.46
5	3.13±0.29	0.48±0.10	55.10±3.22	25.86±0.52	35.33±2.86	26.19±2.81
6	3.55±0.04	0.75±0.06	63.75±2.40	25.18±0.84	37.51±1.05	25.30±3.14
7	4.07±0.16	0.78±0.05	65.66±1.23	23.16±1.36	36.16±2.65	18.87±5.45
8	3.41±0.29	0.88±0.10	59.81±2.48	24.46±0.41	40.02±1.05	22.55±2.43
9	3.54±0.15	0.73±0.10	62.26±1.65	21.87±0.71	40.05±0.70	25.45±8.94
10	3.60±0.05	0.50±0.08	55.36±3.92	25.40±0.72	36.18±2.91	22.96±8.75
11	3.05±0.18	0.50±0.12	63.67±2.56	23.86±2.50	39.09±3.39	29.62±6.78
12	3.42±0.38	0.70±0.08	58.49±2.25	22.36±1.49	35.87±2.78	21.94±3.36
13 (control)	3.05±0.42	0.93±0.10	56.44±1.50	24.41±1.86	36.02±3.61	24.39±1.07
14 (control)	3.27±0.72	0.95±0.05	57.01±3.28	25.54±0.87	37.24±2.01	23.66±1.39
Model	Linear	Quadratic	Cubic	Quadratic	Linear	Linear
*p*-value	0.0055	0.0062	0.4097	0.0388	0.0496	0.0053
R^2^	0.6851	0.8979	0.8895	0.9644	0.4871	0.6878
Adjusted-R^2^	0.6152	0.8128	0.392	0.8769	0.3731	0.6184
Predicted-R^2^	0.3499	0.4733	−22.8968	−1.2748	−0.0356	0.3044
Adeq Precision	8.9203	10.3742	3.962	10.285	4.6499	9.073

Response values represent Means ± SD. L*: brightness, a*: ±red-green, a*: ±red-green, b*: ±yellow-blue. Moisture content, soluble solid content and color experiments were performed in triplicate. Ten replicates of hardness experiments were performed.

**Table 4:** Sensory properties and fitted model statistics of sucrose-free hard candies added with *Cudrania tricuspidata* fruit extract.

**Sample**	Overall Acceptance	Appearance	Flavor	Texture	Color	Clarity	Sweetness	Sourness	Cooling	Hardness	Fracturability	Stickiness
1	4.39±1.87	6.05±1.48	5.30±1.52	4.38±2.32	6.13±1.45	6.08±1.54	5.35±1.49	5.04±1.50	5.29±1.62	3.59±1.84	2.88±1.54	2.68±1.78
2	6.21±1.38	6.01±1.43	5.81±1.40	6.64±1.37	5.96±1.41	6.06±1.49	5.94±1.41	5.40±1.46	5.64±1.55	6.60±1.36	6.61±1.47	6.14±2.05
3	4.29±1.69	5.81±1.49	5.46±1.24	3.85±1.99	5.94±1.50	6.01±1.62	5.63±1.53	5.11±1.59	5.43±1.47	3.51±1.62	3.33±1.92	2.74±2.00
4	3.71±1.87	5.86±1.85	5.50±1.42	3.10±1.92	6.08±1.60	6.56±1.73	5.94±1.28	5.44±1.48	5.49±1.47	3.45±1.82	2.91±1.84	1.83±1.27
5	5.54±1.41	5.43±1.61	5.35±1.43	6.55±1.35	5.99±1.40	5.54±1.53	5.36±1.53	5.20±1.62	5.45±1.65	6.61±1.35	6.53±1.47	5.81±2.04
6	5.18±1.59	5.59±1.60	5.43±1.44	5.38±1.93	5.85±1.58	5.96±1.67	5.69±1.69	5.36±1.62	5.26±1.64	4.41±1.96	4.35±2.27	4.73±2.06
7	5.03±1.75	6.13±1.56	5.71±1.69	4.73±2.23	6.08±1.49	6.10±1.60	6.07±1.41	5.40±1.65	5.78±1.59	3.63±1.73	3.53±1.62	3.43±2.02
8	5.93±1.22	5.81±1.50	5.59±1.46	6.15±1.49	5.96±1.42	5.86±1.37	5.64±1.59	5.33±1.71	5.73±1.59	5.81±1.78	6.03±1.92	5.96±1.85
9	5.59±1.54	5.83±1.49	5.14±1.39	5.94±1.58	5.99±1.37	5.83±1.50	5.36±1.68	5.28±1.68	5.36±1.75	6.23±1.48	6.58±1.48	5.86±2.13
10	5.34±1.64	5.98±1.60	5.46±1.50	5.51±1.89	6.11±1.53	5.80±1.69	5.70±1.51	5.35±1.71	5.84±1.60	4.90±2.27	4.51±2.19	5.20±1.98
11	6.09±1.37	5.94±1.37	5.70±1.48	6.48±1.22	5.98±1.37	6.10±1.27	5.68±1.50	5.38±1.58	5.93±1.70	6.40±1.39	6.71±1.30	6.06±2.02
12	5.36±1.70	6.24±1.43	5.86±1.55	4.95±2.05	6.15±1.32	6.25±1.50	5.89±1.55	5.36±1.64	5.76±1.67	4.28±2.14	3.99±2.20	5.44±1.82
13 (control)	5.90±1.79	5.71±1.56	5.94±1.67	6.36±1.55	5.80±1.63	5.73±1.62	5.64±2.00	5.49±1.57	6.09±1.67	6.31±1.57	6.61±1.48	5.80±1.89
14 (control)	5.67±1.89	5.98±1.50	6.19±1.55	6.11±2.13	6.02±1.60	5.91±1.61	6.00±1.85	5.38±1.77	6.10±1.59	6.41±1.68	6.40±1.74	5.49±2.22
Model	Linear	Cubic	Cubic	Linear	Special Quartic	Linear	Linear	Special Cubic	Cubic	Linear	Linear	Linear
*p*-value	0.0001	0.2987	0.5516	<0.0001	0.0393	0.0319	0.0482	0.0961	0.0331	<0.0001	<0.0001	<0.0001
R^2^	0.8611	0.9242	0.8368	0.9354	0.9547	0.5348	0.4904	0.8069	0.9925	0.8939	0.9161	0.9038
Adj.R^2^	0.8303	0.583	0.1021	0.921	0.834	0.4314	0.3771	0.5753	0.959	0.8703	0.8974	0.8825
Pred.R^2^	0.7286	−14.3311	−43.7979	0.8705	−18.2426	0.158	0.11	−0.802	0.7835	0.8231	0.8672	0.8395
Adeq Precision	11.1297	5.79	3.1224	19.348	8.7539	6.5206	5.1557	6.4978	15.7647	14.6211	15.6455	15.2314

Response values represent Means ± SD. Number of consumer panelists was 80. A 9-point “liking” scale: 1 = extremely dislike, 5 = neither like nor dislike, 9 = extremely like.

**Table 5:** Sensory properties of hard candy with *Cudrania tricuspidata* fruit extract added.

Sensory Property	Samples					
	CF0 ^1^	CF0.25 ^2^	CF0.50 ^3^	CF0.75 ^4^	CF1.00 ^5^	*F*-value
Overall acceptance	5.70±1.63 ^abc^	5.56±1.47 ^ab^	5.95±1.43 ^bc^	6.15±1.48 ^c^	5.29±1.42 ^a^	4.401 ***
Appearance acceptance	6.56±1.65 ^c^	5.75±1.52 ^b^	5.75±1.57 ^b^	5.46±1.67 ^b^	4.83±1.75 ^a^	11.751 ***
Flavor acceptance	5.46±1.48	5.51±1.52	5.83±1.57	5.85±1.67	4.82±1.75	1.104
Texture acceptance	5.90±1.50 ^bc^	5.50±1.55 ^ab^	6.22±1.19 ^c^	6.34±1.27 ^c^	5.27±1.41 ^a^	8.567 ***
Color acceptance	6.43±1.69 ^c^	5.53±1.64 ^b^	5.78±1.62 ^b^	5.41±1.90 ^b^	4.76±1.76 ^a^	9.860 ***
Clarity acceptance	6.57±1.54 ^c^	5.86±1.38 ^b^	6.18±1.41 ^bc^	5.85±1.66 ^b^	5.31±1.40 ^a^	7.884 ***
Sweetness acceptance	5.65±1.89	5.61±1.73	5.90±1.72	6.25±1.70	6.11±1.38	2.168
Sourness acceptance	5.18±1.63	5.13±1.58	5.27±1.50	5.47±1.59	5.06±1.53	0.811
Cooling acceptance	4.97±1.46 ^a^	5.43±1.42 ^ab^	5.46±1.58 ^ab^	5.77±1.58 ^b^	5.61±1.43 ^b^	3.183 **
Hardness acceptance	6.06±1.30 ^b^	5.48±1.45 ^a^	5.96±1.41 ^b^	6.33±1.33 ^b^	5.88±1.51 ^ab^	3.525 **
Fracturability acceptance	5.87±1.36 ^ab^	5.40±1.61 ^a^	5.98±1.36 ^b^	6.18±1.41 ^b^	5.85±1.52 ^ab^	3.062 **
Stickiness acceptance	6.06±1.79 ^c^	5.41±1.95 ^b^	5.42±1.73 ^b^	5.65±1.82 ^bc^	4.78±1.91 ^a^	5.019 ***

Response values represent means ± standard deviations. Number of consumer panelists was 80. ** *p* < 0.01, *** *p* < 0.001. Note: Values followed by different letters in the same column are significantly different (*p* < 0.05) according to Duncan’s test. ^1^ Hard candy added with 0% of *Cudrania tricuspidata* fruit extract. ^2^ Hard candy added with 0.25% of *Cudrania tricuspidata* fruit extract. ^3^ Hard candy added with 0.50% of *Cudrania tricuspidata* fruit extract. ^4^ Hard candy added with 0.75% of *Cudrania tricuspidata* fruit extract. ^5^ Hard candy added with 1.00% of *Cudrania tricuspidata* fruit extract.

## Data Availability

The data presented in this study are available on request from the corresponding author. The data are not publicly available due to the institutional data policy.
